# Imaging patterns of *Lophomonas blattarum* infection in the respiratory tract: a registry-based analysis

**DOI:** 10.1186/s12879-024-09141-2

**Published:** 2024-02-29

**Authors:** Amirmasoud Taheri, Mahdi Fakhar, Ali Sharifpour, Elhamsadat Banimostafavi, Sepideh SafaNavaei, Siavash Abedi, Hossein Mehravaran, Masoud Aliyali, Ahmad Shafahi, Asieh Delpzir

**Affiliations:** 1 Toxoplasmosis Research Center, Communicable Diseases Institute, Iranian National Registry Center for Lophomoniasis (INRCL), Mazandaran University of Medical Sciences, Farah-Abad Road, Sari, P.O Box: 48471-91971, Iran; 2 Pulmonary and Critical Care Division, Imam Khomeini Hospital, Mazandaran University of Medical Sciences, Sari, Iran; 3 Radiology Department, Iranian National Registry Center for Lophomoniasis (INRCL), Mazandaran University of Medical Sciences, Sari, Iran; 4 Department of Internal Medicine, CardiovascularResearch Center, Institute of Basic and ClinicalPhysiology Sciences, Kerman University ofMedical Sciences, Kerman, Iran

**Keywords:** *Lophomonas blattarum*, Chest CT scan, Paranasal sinuses CT scan, Lophomoniasis, Radiologic findings

## Abstract

**Background:**

*Lophomonas blattarum* is an emerging protozoan that mostly infects the lower respiratory tract and causes pulmonary lophomoniasis. Radiologic findings in patients with pulmonary lophomoniasis have yet to be studied. Thus, we conducted a registry-based clinical investigation to evaluate the radiologic findings of lophomoniasis.

**Methods:**

In this cross-sectional study, 34 *Lophomonas* positive patients were enrolled. Demographic data, relevant characteristics, and radiologic findings of the patients were recorded and analyzed.

**Results:**

Thirty-four (male = 18, female = 16) patients with an average age of 52.21 ± 20.48 years old were examined. Radiological findings such as Alveolar consolidation (26.5%), Ground glass opacity (5.9%), Centrilobular nodules (23.5%), Tree -in- bud (38.2%), Cavitation (23.5%), Pleural effusion (23.5%), Interstitial opacity (8.8%), Lymphadenopathy (23.5%), Bronchocele (5.9%), Bronchiectasis (29.4%), Nodules (8.8%) and Mass (11.8%) were obtained, that the frequency of all radiological findings was less than 50%.

**Conclusion:**

In this study, the most common radiological findings in patients with lophomoniasis were tree-in-bud nodules, alveolar consolidation, bronchiectasis, and centrilobular nodules which were mostly seen in the right lung and its middle and lower lobes. Given that the radiologic findings of this disease are unknown, it can be considered in differential diagnosis.

## Introduction

*Lophomonas blattarum* is an endocommensal flagellated protozoan parasite of the order *Hypermastigidia* and the suborder *Lophomonadia*, found in the hindgut of various arthropods such as termites and cockroaches [1]. This protozoon’s cysts can enter the environment via insect feces and infest human organs via respiratory aerosols [2]. This parasite can infect a variety of tissues and organs, including the maxillary and other sinuses, lungs, respiratory tract, and reproductive system, potentially causing irreparable complications such as a pulmonary cavity [3, 4]. The most common symptoms are fever, cough, and mucus secretions up to respiratory failure. Therefore, based on clinical findings and laboratory tests, it is difficult to distinguish them from other common diseases with similar symptoms such as pneumonia and bronchitis [5, 6]. *Lophomonas* is diagnosed using one or more of the following techniques: nasal discharge smear, bronchoscopic brush smear, bronchoscopic biopsy smear, and bronchoalveolar lavage(BAL) [7]. Imaging findings in lophomoniasis may reveal features of pneumonia, bronchiectasis, lung abscess, and pleural effusion. Because of its resemblance to epithelial cells, it cannot be seen under the light microscope and is easily overlooked [8]. Meanwhile, with a precise and prompt diagnosis, metronidazole provides an effective treatment [1, 9]. Given that the diagnosis of this protozoan is based on a BAL sample obtained by bronchoscopy, which is an invasive procedure, and no study has been performed on the imaging findings of this protozoan, we designed a study in Iran where the epidemiology of *Lophomonas* is nearly 23% [10], to support the diagnosis of this disease by examining the imaging features of this infection.

## Materials and methods

This was a pilot descriptive cross-sectional study conducted in 2021 at Imam Khomeini Hospital, Sari, Iran. The target group of the study are patients infected with *Lophomonas* who have confirmed the diagnosis of this parasite and registered in the Iranian National Registry Center for Lophomoniasis (INRCL). Inclusion criteria were *Lophomonas* positive patients, which is diagnosed by specific polymerase chain reaction method, and had chest and paranasal sinus CT scans. After meeting the inclusion criteria, 34 patients entered in our study. Demographic information of all identified patients, including age, gender, and underlying disease, as well as chest CT scan findings, affected side and lung lobe findings, and paranasal sinus CT scan findings were recorded in a predesigned checklist. To assess the dependency of qualitative variables, the chi-square test was used and a p-value less than 0.05 was considered significant. All data were analyzed with SPSS version 22.

## Results

Fifty-nine *Lophomonas* positive patients were registered in the INRCL, however, only the radiological features of 34 (F = 16(47.1%), M = 18 (52.9%)) of them were available. The ages ranged from 5 to 82, with an average of 52.21 ± 20.48. In addition, 19 patients (55.9%) had a concomitant underlying disease, demonstrating the importance of the underlying disease in the development of this infection.

Of the 19 patients with underlying disease, 4 (11.8%) had bronchogenic carcinoma, and one of these patients had both bronchogenic carcinoma and tuberculosis (TB) infection. Five patients (14.7%) had *Mycobacterium tuberculosis* infection, four patients (11.8%) had diabetes, and three patients (8.8%) received corticosteroids metered dose inhaler for asthma or bronchiolitis. Five other individuals had Hodgkin’s lymphoma, sarcoidosis, a history of kidney transplantation, chronic obstructive pulmonary disease (COPD), and a lung hydatid cyst (Table 1).

**Table 1:** Demographic findings and characteristics of patients with *Lophomonas* infection referred to INRCL.

Characteristic	Mean ± SD/ Frequency (%)
**Age**	52.2 ± 20.8
**Gender**	
Male	18(52.9)
Female	16(47.1)
**Underlying disease**	
Tuberculosis	5 (14.7)
Bronchogenic carcinoma	4 (11.8)
Diabetes	4 (11.8)
Hodgkin lymphoma	1 (2.9)
Use of corticosteroid spray	3 (8.8)
Sarcoidosis	1 (2.9)
Kidney transplant	1 (2.9)
Chronic obstructive airways disease (COPD)	1 (2.9)
Lung hydatid cyst	1 (2.9)

**Fig. 1:**
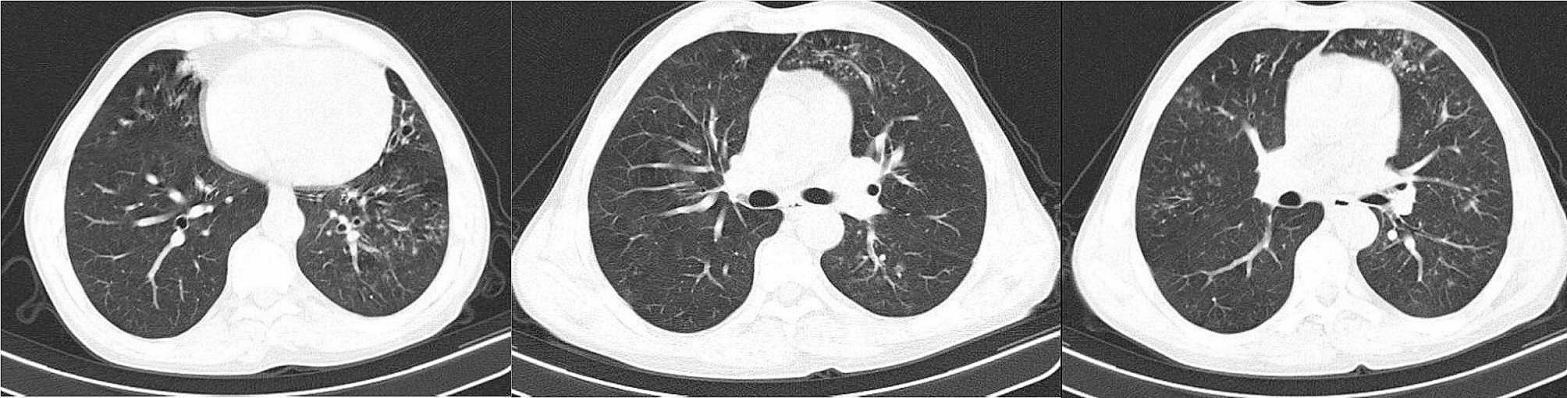
Lung CT scan of 45 years old man with lophomoniasis. Tree-in-bud in right upper lobe (RUL), right middle lobe (RML), right lower lobe (RLL), left upper lobe (LUL), lingula, right upper lobe (LLL) and bronchiectasis in lingula

**Fig. 2:**
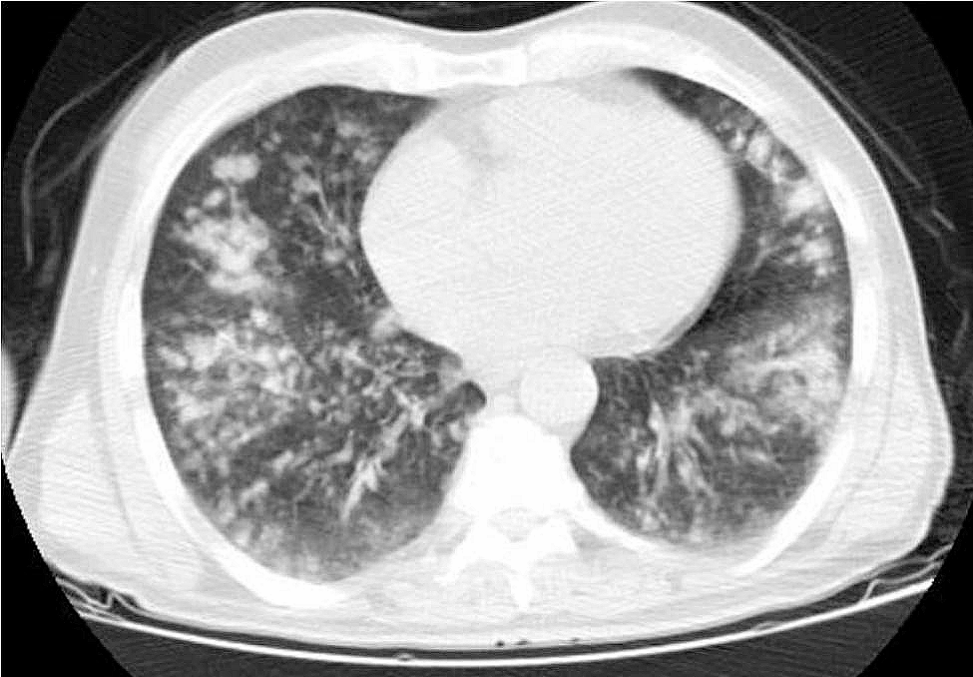
Lung CT scan of a 71-year-old man with concomitant TB. Centrilobular nodules and lobular consolidation in both lungs

CT scans of some patients are demonstrated below. Figure 1 depicts the CT image of the lungs of a 45-year-old individual with a kidney transplant history. And Fig. 2 shows lung CT scan of a 71-year-old man with concomitant TB.

Figure 2 shows a 71-year-old man with co-existing tuberculosis.

**Fig. 3:**
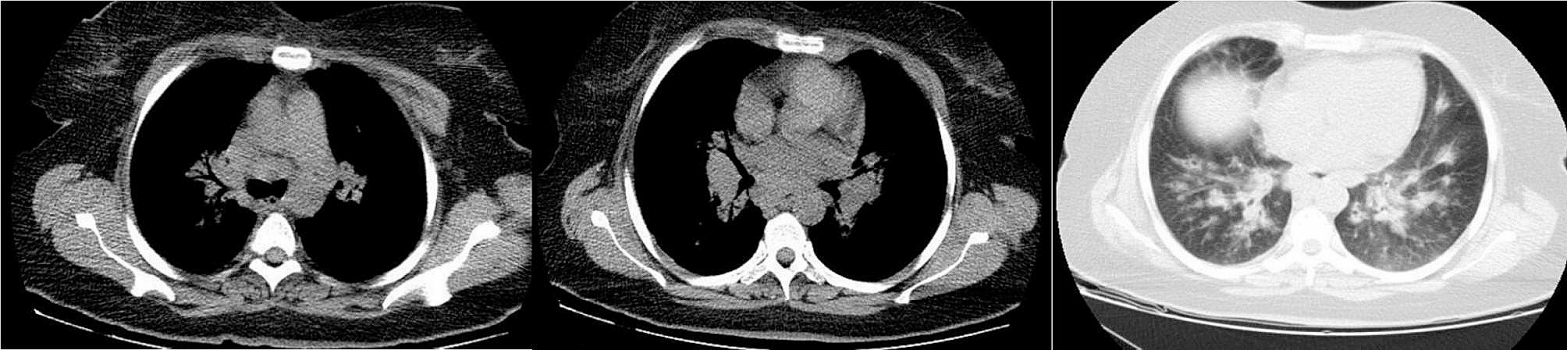
Lung CT scan of a 43-year-old woman with simultaneous histological diagnosis of sarcoidosis. Mediastinal and hilar lymphadenopathy. Diffuse peribronchovascular nodules and parahilar confluent consolidation

The lung CT scan of a 43-year-old woman with a simultaneous histological diagnosis of sarcoidosis after bronchoscopy is shown in Fig. 3.

**Fig. 4:**
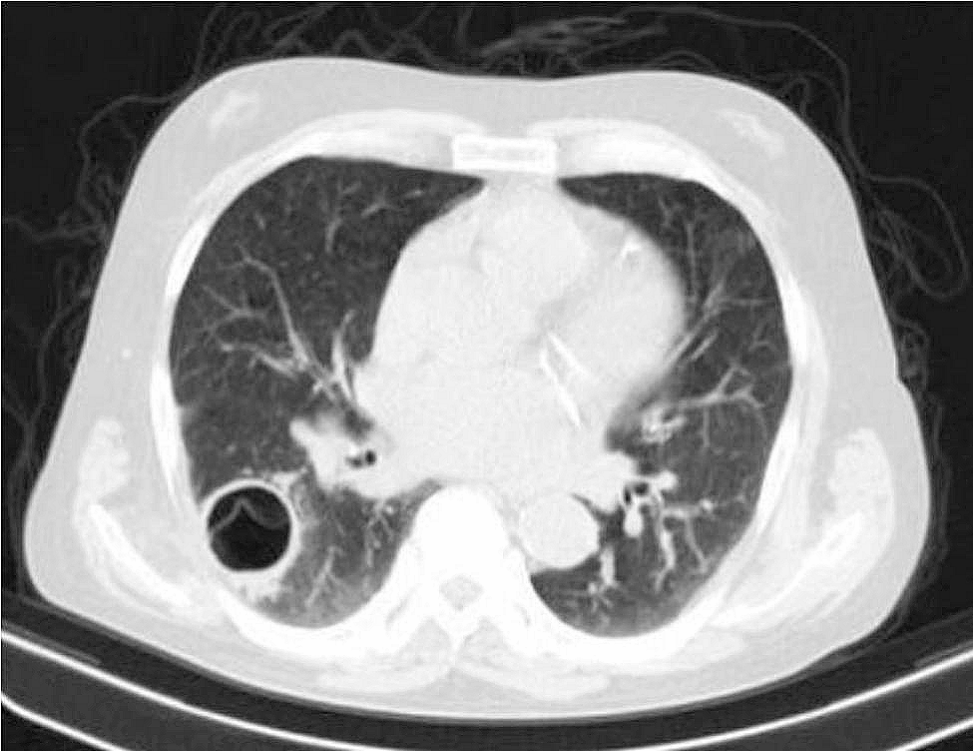
Lung CT scan of a 40-year-old man with concomitant hydatid cyst. Two cavitary lesions with internal floating membrane with peripheral alveolar opacification

Figure 4 shows a lung CT scan of a 40-year-old man with a concomitant hydatid cyst.

**Fig. 5:**
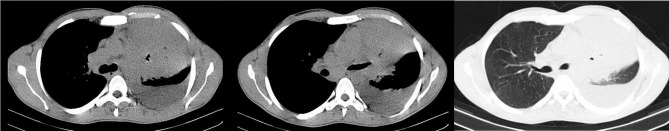
Lung CT scan of a 36-year-old man with concurrent bronchogenic carcinoma of the Left hilar mass with pleural effusion and mediastinal lymphadenopathy

The lung CT scan of a 36-year-old man with concurrent bronchogenic cancer was demonstrated in Fig. 5.

**Fig. 6:**
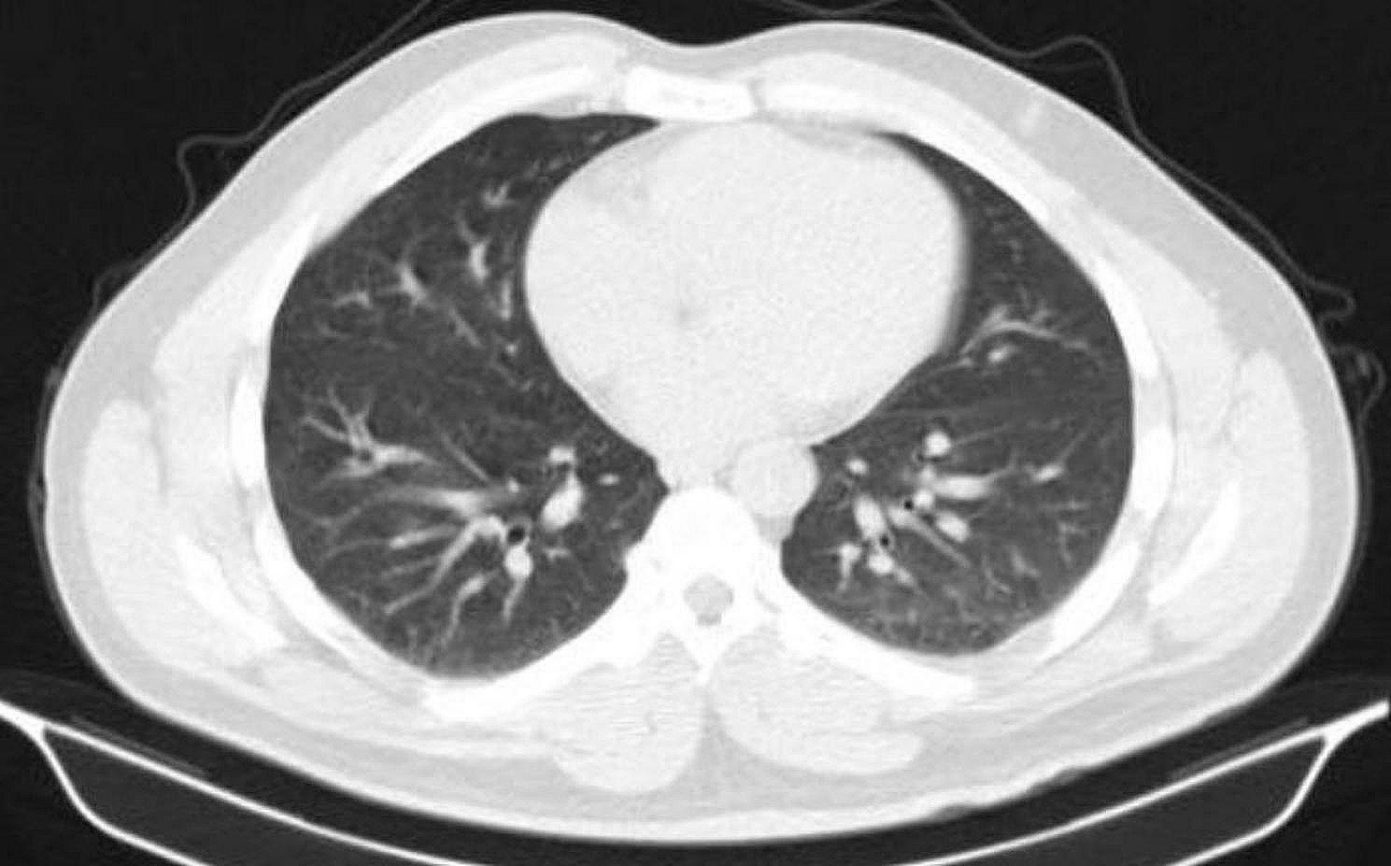
Lung CT scan of a 38-year-old man without underlying disease, normal

Figure 6 shows a lung CT scan of a patient with no underlying disease.

A chest CT scan revealed no abnormalities in four patients who complained of coughing, sputum, and wheezing. Alveolar consolidation (26.5%), ground glass opacity (9.5%), centrilobular nodules (5.23%), Tree-in-bud (2.38%), cavitation (5.23%), pleural effusion (23.5%), interstitial opacity (8.8%), lymphadenopathy (23.5%), bronchocele (5.9%), bronchiectasis (29.4%), nodule (8.8%) In other patients, the frequency of all radiological findings was less than 50%, as shown in Table 2.

**Table 2:** Radiological findings of patients with lophomoniasis

Variables	Frequency (percentage)	*P*-value
Alveolar consolidation	9 (26.5%)	0.812
Ground glass opacity	2 (5.9%)	
Centrilobular nodules	8 (23.5%)	
Tree-in-bud	13 (38.2%)	
Cavitation	8 (23.5%)	
Pleural effusion	8 (23.5%)	
Interstitial opacity	3 (8.8%)	
Lymphadenopathy	8 (23.5%)	
**Anatomical location**	12 (35.3%) upper right	
	15 (44.1%) middle right	
	15 (44.1%) lower right	
	13 (38.2%) upper left	
	15 (44.1%) lower left	
	8 (23.5%) lingola	
Bronchocele	2 (5.9%)	
Bronchiectasis	10 (29.4%)	
Nodules	3 (8.8%)	
Mass	4 (11.8%)	

Furthermore, only one kidney transplant patient complained of a history of upper airway involvement in the form of nasal congestion in addition to symptoms of lower airway involvement and was evaluated for the paranasal sinuses by CT scan of the paranasal sinuses and a slight increase in the thickness of the mucosa in the sinuses. Paranasal infections have been observed; however, due to the positive results of *Lophomonas* in the evaluation of the BAL of the patient and the patients who initially received broad-spectrum antibiotics, examination of the smear or culture of the paranasal sinus secretions for *Lophomonas* was not performed; thus, the involvement of sinus with *Lophomonas* infection in this patient cannot be confirmed. However, there were no other cases of upper airway involvement and CT scan interpretation of the paranasal sinuses in the other patients examined in this study.

Table 3 shows the results of the radiological findings by gender. The current study found that bronchiectasis is more common in men. There was no significant association between other radiological findings and the gender variable (*P* = 0.04). However, in relation to the anatomical location of the radiological findings based on gender, it was found that males had a greater involvement of the right upper lobe of the lung, which was statistically significant (*P* = 0.001).

**Table 3:** Results of radiological findings based on gender in patients with lophomoniasis

Variables	Male	Female	*P*-value	Odds Ratio
Alveolar consolidation	6(33.3%)	3(8.18%)	0.336	2.167
Ground glass opacity	0(0%)	2(12.5%)	0.122	0.438
Centrilobular nodules	3(16.7%)	5(31.3%)y	0.317	0.440
Tree-in-bud	5 (27.8%)	8(50%) y	0.183	0.385
Cavitation	5(8.27%)	3(18.8%)	0.536	1.667
Pleural effusion	5(27.8%)	3(8.18%)	0.536	1.667
Interstitial opacity	2(11.1%)	1(6.3%)	0.618	1.875
lymphadenopathy	6(33.3%)	2(12.5%)	0.153	3.5
**Anatomical site**				
upper right	11(61.1%)	1(6.3%)	0.001	23.571
middle right	9(50%)	6(37.5%)	0.464	1.667
lower right	9(50%)	6(37.5%)	0.464	1.667
upper left	9(50%)	4(25%)	0.134	3.000
lower left	8(44.4%)	7(43.8%)	0.968	029.1
lingola	5(27.8%)	3(18.8%)	0.536	1.667
bronchocele	1(5.6%)	1(6.3%)	0.932	0.882
bronchiectasis	2(12.5%)	8 (44.4%)	0.041	5.600
nodules	2(11.1%)	1(6.3%)	0.618	1.875
mass	4(22.2%)	0(0%)	0.045	0.467

y means: Yes

Table 4 presents the outcomes of radiological findings based on age. The current study’s findings revealed that there was no significant variation in radiological findings based on age. The anatomical position of the radiological results was analyzed based on the age of patients less than, equal to, and older than 60 years, and it was found that patients 60 years and older had a greater involvement of the lower lobe of the right lung, which was statistically significant (*P* = 0.001).

**Table 4:** The results of the radiological findings based on the age variable in patients with lophomoniasis

Variables	Under 60 years	Over 60 years	*P*-value	Odds Ratio
Alveolar consolidation	4 (23.5%) y	5(29.4%) y	0.697	1.354
	13 (76.5%) *n*	12 (70.6%) *n*		
Ground glass opacity	1(5.9%) y	1(5.9%) y	1	1
	16 (94.1%)*n*	16 (94.1%)*n*		
Centrilobular nodules	2 (11.8%)y	6(35.3%) y	0.106	4.094
	15 (88.2%) *n*	11(64.7%) *n*		
Tree-in-bud	8(47.1%) y	5(29.4%) y	0.290	0.469
	9(52.9%) *n*	12(70.6%) *n*		
Cavitation	2 (11.8%)y	6 (35.3%)y	0.106	4.091
	15 (88.2%) *n*	11 (64.7%) *n*		
pleural effusion	2 (11.8%)y	6 (35.3%)y	0.106	4.091
	15 (88.2%) *n*	11 (64.7%) *n*		
Interstitial opacity	1 (5.9%) y	2 (11.8%) y	0.545	2.133
	16 (94.1%) *n*	15 (88.2%) *n*		
lymphadenopathy	2 (11.8%)y	6 (35.3%)y	0.106	4.091
	15 (88.2%) *n*	11 (64.7%) *n*		
**Anatomical site**				
upper right	4 (23.5%)	8(47.1%)	0.151	2/889
middle right	10(58.8%)	5(29.4%)	0.084	3.429
lower right	2 (11.8%)	13(76.5%)	0.000	24.375
upper left	6 (35.3%)	7 (41.2%)	0.724	1283
lower left	6 (35.3%)	9 (52.9%)	0.300	2.063
lingola	2 (11.8%)	6 (35.3%)	0.106	4.091
bronchocele	1 (5.9%)y	1 (5.9%)y	1.000	1.000
	16 (94.1%)*n*	16 (94.1%)*n*		
bronchiectasis	5 (29.4%)y	5 (29.4%)y	1.000	1.000
	12 (70.6%)*n*	12 (70.6%)*n*		
nodule	1 (5.9%)y	2 (11.8%)y	0.545	2.133
	16 (94.1%)*n*	15 (88.2%) *n*		
mass	2 (11.8%)y	2 (11.8%)y	1.000	1.000
	15 (88.2%) *n*	15 (88.2%) *n*		

y means:Yes; n means: No

Table 5 shows the outcomes of radiological findings based on the underlying medical history. The current study found that lymphadenopathy was more likely in patients with a history of underlying disease (*P* = 0.039). However, there was no significant association between the anatomical location of the observations and the underlying medical history.

**Table 5:** The results of the radiological findings based on the underlying disease history in patients with lophomoniasis

Variables		Having underlying disease	Does not have underlying disease	*P*-value	Odds Ratio
Alveolar consolidation	Presence	7 (36.8%)	2 (13.3%)	0.123	3.792
	Absence	12 (63.2%)	13 (86.7%)		
Ground glass opacity	Presence	1 (5.3%)	1 (6.7%)	0.863	0.778
	Absence	18 (94.7%)	14 (93.3%)		
centrilobular nodules	Presence	5 (26.3%)	3 (20%)	0.666	1.42
	Absence	14 (73.7%)	12 (80%)		
Tree-in-bud	Presence	5 (26.3%)	8 (53.3%)	0.107	0.313
	Absence	14 (73.7%)	7 (46.7%)		
cavitation	Presence	5 (26.3%)	3 (20%)	0.666	1.429
	Absence	14 (73.7%)	12 (80%)		
pleural effusion	Presence	6 (31.6%)	2 (13.3%)	0.213	3.000
	Absence	13 (68.4%)	13 (86.7%)		
Interstitial opacity	Presence	3 (15.8%)	0 (0%)	0.107	0.516
	Absence	16 (84.2%)	15 (100%)		
Lymphadenopathy	Presence	7 (36.8%)	1 (6.7%)	0.039	8.167
	Absence	12 (63.2%)	14 (93.3%)		
**Anatomical location**					
upper right		9 (47.4%)	3 (20%)	0.097	3.600
middle right		10 (52.6%)	5 (33.3%)	0.260	2.222
lower right		9 (47.4%)	6 (40%)	0.667	1.350
upper left		3 (23.1%)	5 (33.3%)	0.601	1.455
lower left		9 (47.4%)	6 (40%)	0.667	1.350
lingola		6 (31.6%)	2 (13.3%)	0.213	3.000
bronchocele	Presence	1 (5.3%)	1 (6.7%)	0.863	0.778
	Absence	18 (94.7%)	14 (93.3%)		
bronchiectasis	Presence	7 (36.8%)	3 (20%)	0.285	2.333
	Absence	12 (63.2%)	12 (80%)		
nodules	Presence	3 (15.8%)	0 (0%)	0.107	0.516
	Absence	16 (84.2%)	15 (100%)		
mass	Presence	4 (21.1%)	0 (0%)	0.059	0.500
	Absence	15 (78.9%)	15 (100%)		

The radiological findings revealed nodules in three patients, all with underlying disease. One patient had sarcoidosis, one had lung cancer and concomitant TB, and one had pulmonary emphysema. A tumor was also seen in four patients, all had lung cancer, and one had concurrent TB infection. Furthermore, lymphadenopathy was observed in eight patients, with only one case having no history of underlying disease; five cases with concurrent underlying disease included Hodgkin’s, lymphoma, sarcoidosis, lung cancer and TB; lymphadenopathy in these patients can be caused by an underlying disease. Cavity was also found in eight patients, four of whom had TB and one had hydatid cysts, and three who had no concomitant underlying disease. Furthermore, pleural effusion was observed in eight individuals, six of whom had concomitant underlying disease, three cases of lung cancer, one case of TB, and two cases of diabetes, so the imaging findings listed above comprise nodule, mass, lymphadenopathy, cavity, and pleural effusion. It appears that it can be caused by an underlying condition in some patients.

## Discussion

*Lophomonas blattarum* is an endocommensal flagellated protozoan parasite primarily found in the hindgut of various arthropods, such as termites and cockroaches [11]. Although its primary hosts are insects, this protozoon has the potential to infect humans when its cysts are inhaled as respiratory aerosols derived from insect feces [12]. Once in the human body, *Lophomonas* can invade various tissues and organs, leading to a range of clinical manifestations, including respiratory symptoms, such as fever, cough, and mucus secretion, which can progress to severe respiratory failure [13].

This cross-sectional study aimed to investigate the radiological features of *Lophomonas* infection in patients who had confirmed diagnoses of this parasitic infection and underwent chest and paranasal sinus CT scans. A total of 34 patients with positive *Lophomonas* results in the INRCL were included in the study. The findings of this study shed light on the radiological manifestations of *Lophomonas* infections and provided valuable insights into the disease’s impact on different anatomical locations of the respiratory system.

The results of the study revealed a wide range of radiological findings in patients with *Lophomonas* infection. The most common radiological abnormalities included alveolar consolidation, ground-glass opacity, centrilobular nodules, and tree-in-bud appearance. In a case report from China, a 21-year-old patient presenting with symptoms of productive cough and fever was diagnosed with *Lophomonas* based on the findings from CT scan wich revealed ground-glass opacity, patchy consolidation and patchy or streaky shadows distributed in bilateral lungs [14].

It is noteworthy that some of the patients in this study had concomitant underlying medical conditions. This is particularly relevant because some radiological findings, such as lymphadenopathy, cavitation, pleural effusion, nodules, and masses, were more likely to be observed in patients with underlying diseases. These underlying conditions might influence the clinical manifestations of *Lophomonas* infection and contribute to the heterogeneity of the radiological presentations observed in this study.

Interestingly, the study also highlighted gender-related differences in the radiological manifestations of *Lophomonas* infection. Bronchiectasis was more common in male patients, and males had a greater involvement of the right upper lobe of the lung. While the reasons for these gender-related differences remain unclear, they warrant further investigation to better understand the potential impact of gender on *Lophomonas* infection outcomes and presentations.

Moreover, age-related variations were explored, but no significant differences were found in radiological findings among different age groups. However, patients aged 60 and older showed a statistically significant association with greater involvement of the lower lobe of the right lung. Age-related changes in the respiratory system and potential interactions between age and *Lophomonas* infection may have contributed to these observations.

The study has some limitations that should be acknowledged. The sample size was relatively small, and the data were obtained from a single center. Additionally, the study design was cross-sectional, limiting the ability to establish causal relationships between *Lophomonas* infection and specific radiological findings. Further prospective studies with larger sample sizes and multiple centers are warranted to validate these findings and explore potential risk factors associated with specific radiological manifestations of *Lophomonas* infection.

It should be noted that due to being a single-center study we might face selection bias and thus findings of a single-center study may not be directly applicable to other populations or healthcare settings due to potential variations in demographics, cultural factors, and healthcare practices.

In conclusion, this cross-sectional study provides valuable insights into the radiological features of *Lophomonas* infection in human patients. The diverse and non-specific imaging presentations observed in this study emphasize the importance of considering *Lophomonas* infection in the differential diagnosis of respiratory symptoms, especially in regions where the parasite is prevalent. Moreover, the findings highlight the potential impact of underlying medical conditions and gender on the radiological manifestations of *Lophomonas* infection. Increased awareness among healthcare professionals about the imaging characteristics of *Lophomonas* infection may lead to earlier and more accurate diagnoses, facilitating prompt and effective treatment interventions, and ultimately improving patient outcomes.

## Data Availability

The data are available to the correspondence author and can be reached on request.

## Declarations

### Abbreviations

Iranian National Registry Center for Lophomoniasis (INRCL),

broncho alveolar lavage (BAL).

computed tomography (CT).

### FUNDIG

The authors did not receive support from any organization for the submitted work.
